# Medical students' knowledge, attitude, and practice regarding hepatitis B and C virus infections in Jordan: A cross‐sectional study

**DOI:** 10.1002/hsr2.70150

**Published:** 2024-12-11

**Authors:** Husam Abu Suilik, Osama Alfaqeh, Abdalqader Alshrouf, Omnia M. Abdallah, Jehad Feras AlSamhori, Lujain Lataifeh, Mohammad Alsbou, Mohammad Abusuilik, Nawar Khalil, Mohand Odeh, Hashem Abu Serhan, Abdulqadir J. Nashwan, Anas Bani Hani

**Affiliations:** ^1^ Faculty of Medicine The Hashemite University Zarqa Jordan; ^2^ Faculty of Science, Microbiology Department Ain Shams University Cairo Egypt; ^3^ Faculty of Medicine University of Jordan Amman Jordan; ^4^ Faculty of Medicine Jordan University of Science and Technology Irbid Jordan; ^5^ Faculty of Medicine Yarmouk University Irbid Jordan; ^6^ Department of Clinical Pharmacy and Pharmacy Practice, Faculty of Pharmaceutical Sciences The Hashemite University Zarqa Jordan; ^7^ Department of Ophthalmology Hamad Medical Corporations Doha Qatar; ^8^ Nursing & Midwifery Research Department (NMRD) Hamad Medical Corporation Doha Qatar

**Keywords:** hepatitis B & hepatitis C, Jordan, medical students

## Abstract

**Background and Aims:**

We aimed to assess the levels of hepatitis B and C knowledge, attitudes, and practices among medical students in Jordan. A survey included participation from medical students across all faculties in Jordan, from 2022 to 2023.

**Methods:**

The data were analyzed with The R Statistical Software (v4.1.2; R Core Team 2022) using descriptive statistics, and multivariate regression analyses. A *p* ≤ 0.05 was considered statistically significant.

**Results:**

In this study of 2602 participants, the average age was 21.4, with 52.6% females. Most were fourth‐year medical students (19.1%), and 52.3% were in clinical years. The mean knowledge score was 14 (SD ± 2.5) out of 20, categorized as high at 58.84%, and low at 41.16%. The mean practice score was 4.89 (SD ± 1.1) out of 7, with good practice in 65%, and low in 35%. The mean attitude score was 1.6 (SD ± 3.1), categorized as low in 81.7%, and high in 18.3%,. High levels of KAP were associated with gender, year of study, and university. Male respondents had lower knowledge (OR: 0.73; *p*: 0.001; 95% CI: −0.50 to −0.13), and students in the first to fifth years scored lower than sixth‐year students. There were moderate positive associations between knowledge and attitude (*r*: 0.33, *p* < 0.001), and weak positive associations between knowledge and practice (*r*: 0.17, *p* < 0.001), and attitude and practice (*r*: 0.133, *p* < 0.001).

**Conclusion:**

In conclusion, the participants revealed high adherence to some practices and intermediate knowledge levels. Gender, academic year, and university affiliation emerged as significant factors, highlighting the necessity for tailored interventions.

## 1 INTRODUCTION

The viral hepatitis pandemic has severely impacted people's lives, communities, and healthcare systems. Chronic hepatitis B virus (HBV) and hepatitis C virus (HCV) are present in approximately 240 million and 130–150 million people worldwide, respectively. Their annual death toll is estimated to be 1.4 million due to acute infection, hepatitis‐related liver cancer, and cirrhosis. Hepatitis B and C viruses are responsible for about (47% and 48%) of those fatalities, respectively. (*World Health Organization. Global Hepatitis Report 2017*).^1^ *‏* The national prevalence of HBV in Jordan dropped from (9.9%) in 1985 to (2.4%) in 2016 after the introduction of the HBV vaccination program for newborns and high‐risk groups.^2^

Over the past 30 years, the implementation of an expanded immunization program for infants and adolescents, alongside improved awareness among the general population and a better understanding of the socioeconomic landscape of the country, has contributed significantly to the substantial reduction in the prevalence of HBV infection in Jordan.^3^ Healthcare workers (HCWs) are at a high risk of contracting bloodborne infections due to the particular problems presented by Jordan's healthcare landscape. Since they are more prone than other HCWs to catch these infections, this is especially important for students and HCWs who are still in their training. The need for better training and prevention measures is evident given that sharps injuries account for over 40% of HCV infections among HCWs. Prüss‐Üstün et al.,^4^

By 2030, the World Health Organization hopes to eradicate viral hepatitis. (*World Health Organization. Global Health Sector Strategy on Viral Hepatitis 2016‐2021. Towards Ending Viral Hepatitis. No. WHO/HIV/2016.06*).^5^ *‏* Acquiring more knowledge is one of the critical measures in eliminating viral hepatitis.^6^ Educational initiatives are required to improve knowledge and awareness. Before starting these educational activities, we must accurately assess the existing state of the target group's knowledge, attitude, and practice (KAP) regarding hepatitis. Medical students exhibited suboptimal levels of knowledge, attitudes, and practices in understanding the risk of HBV and HCV infections and prevention strategies. Karimi‐Sari et al.,^7^ Moreover, this remains a significant issue in many nations' healthcare systems, which raises the risk of infections. Successful public health guidelines and approaches will significantly decrease and improve the HBV situation in any society.^8^

A cross‐sectional study conducted in Jordan^9^ showed inadequate attitudes and knowledge regarding HBV with an acceptable level of HBV practice, but not focusing on the KAP related to hepatitis C, and excluding medical students from the basic years. Also, several studies have assessed the knowledge, attitudes, and practices regarding the risk of hepatitis B and C infection and control measures among university students^7^, ^10^, ^11^, ^12^ however, none of them focused on all medical students in Jordan. Even though Jordan's HBV infection prevalence has significantly decreased over the previous 30 years, Effective public health interventions require an understanding of the current status of hepatitis B and C medical education in Jordan. Hence, this study assesses the knowledge, attitudes, and practices regarding hepatitis B and C infection and control measures among all medical students in Jordan. This study may provide insight into improving control measures among medical students for better outcomes, avoiding future hepatitis B and C infections and their complications, and improving the overall quality of life among medical students in Jordan.

## 2 MATERIALS AND METHODS

### 2.1 Study population

The sample was collected from the March 1, 2023 to the July 1, 2023 from medical students at the six universities in Jordan. All students from the first to the sixth years were eligible while students in their internship year were excluded. No other inclusion/exclusion criteria were applied. The data was collected by an online survey that was created on the Google Forms website and posted on social media sites like Facebook, Telegram, and WhatsApp. In addition, a supervisor with access to the Google Form website served as a mentor for the data collection team. Data collection from respondents used convenience technique. To calculate the sample size, we supposed that about half of the students (with a 5% margin of error) would have poor knowledge, attitude, and practice, which generated a sample size of 385, based on a 95% confidence interval (95% CI).

### 2.2 Instrument design

We developed a self‐administered close‐ended online questionnaire in English, based on previous research that had been conducted in Iran and Vietnam,^7^, ^13^ Our study aimed to meet fundamental requirements rather than creating a predictive or assessment tool. We focused on achieving face and content validity, which was validated by consultations with ten gastroenterologists and infectious disease specialists. Validity was assessed using face validity and the content validity ratio as suggested by Lawshe.^14^ During this process, one question was removed, and three were modified. We then piloted the revised 38‐item questionnaire with 30 subjects to ensure its suitability for Jordanian students; these subjects were excluded from the final analysis. The questionnaire assessed demographics, knowledge, attitudes, and practices. Specifically, the questionnaire included four demographic variables: age, gender, academic year, and the university of medicine. The knowledge section consisted of 20 yes/no questions about HBV and HCV infections, covering prevalence, transmission, risk, preventative measures, screening procedures, and treatment methods. The attitudes regarding hepatitis. Regarding awareness, stigma, and perceived severity of hepatitis B and C, among other things, participants' subjective feelings, opinions, and assessments are referred to as “attitude toward hepatitis.” The attitudes toward hepatitis section included seven questions in ordinal categories of strongly agree, agree, no idea, disagree, and strongly disagree. Each question's grade ranged from “+2 to –2” if the statement was accurate and “–2 to +2” if the statement was incorrect. Additionally, the survey asked questions about the participants' approaches to hepatitis B and C control and prevention. In this study, “level of practice” refers to the self‐reported measures and practices that participants have taken to manage and prevent infections with hepatitis B and C. 7 yes/no questions about preventive and treatment strategies were included in the medical practices section. In the knowledge and practice sections, a score of 1 (one) was assigned for every correct answer, whereas an incorrect response was given a score of 0 (zero) and there was no deductible mark for a wrong answer. The maximum combined score reached 7 points for knowledge and 20 points for practice. The total score for the knowledge and practice sections was characterized using the mean and standard deviation, while the attitude score was computed by aggregating individual results from the attitude test. The potential score range for the knowledge section was 0 to 20. We use a 70% cut point similar to the previous study.^9^ It was categorized as “high knowledge” if the score exceeded 14, and “low knowledge” if it was less than 14. For the practice section, scores ranged from 0 to 7, and they were categorized as “good practice” if they were above 5, and “poor practice” if they were less than 5. Attitude scores spanned from −14 to +14, with classifications of “high attitude” if they exceeded 5, and “low attitude” if they were less than 5.

### 2.3 Ethical considerations

The study was conducted following the Declaration of Helsinki guidelines and approved by the Institutional Review Board (IRB) at the Hashemite University in Zarqa, the Hashemite Kingdom of Jordan on 26 December 2022 in meeting No. 2200834. Written informed consent has been obtained from students. Only those who consented to take part in the study were included. We assured the students that they were free to participate in or withdraw from the study, and their participation would not affect their academic performance. The study did not include any incomplete questionnaires.

### 2.4 Statistical analysis

The data were collected by Google Forms and then extracted and stored in a Microsoft Excel sheet. The R Statistical Software (v4.1.2; R Core Team 2022) was utilized to analyze the data. All quantitative variables were checked for normality by a one‐sample Kolmogorov‐Smirnoff test to assess the normality of the data distribution. The data distribution was non‐parametric if the test's significant value was higher than 0.05. In this study, the value was less than 0.05. Then, the Mann‐Whitney and Kruskal‐Wallis tests were used to compare the quantitative variables, Mann‐Whitney was applied for the quantitative bivariate, and Kruskal‐Wallis tests were applied for the multivariate. Chi‐square tests were used to analyze the associations between KAP and demographic factors. The Spearman test examined the partial correlation between the total scores of the three domains KAP. The significance level for P values was set at 0.05 are two‐sided. The regression analysis was conducted to detect the effects of variables on the total domain's score and calculate the odds ratio (OR) of the predictors. Univariate and multivariate Logistic regression analysis examined the participants' characteristics and knowledge, attitudes, and practices towards hepatitis. ORs with their 95% CIs and *p* values were reported, and the skewed data was transformed to apply the logistic regression.

## 3 RESULTS

### 3.1 Demographic

A total of 2602 participants completed the questionnaire. The mean age of participants was 21.4 (SD ± 1.8) with 1368 (52.6%) being female and the remaining 1234 (47.4%) being male. Among the respondents, fourth‐year medical students had the highest representation, with 497 participants (19.1%) and first‐year students were the lowest 387 (14.9%). When comparing the year of study, it was found that 1,361 participants (52.3%) were in clinical years (fourth, fifth, and sixth), while the rest were in preclinical years (first, second, and third) (47.7%). Medical students from Hashemite University and Mut'ah University constituted the largest group, accounting for (17.6%) of the participants. Table 1 demonstrates the demographic characteristics of the study participants.

**Table 1:** Demographic characteristics of study participants.

Variable	Total
Total	2602 (100)
Age	21.4 ± 1.8
Gender	
Female	1368 (52.6)
Male	1234 (47.4)
Academic Year	
1	387 (14.9)
2	462 (17.8)
3	392 (15.1)
4	497 (19.1)
5	468 (18.0)
6	396 (15.2)
University	
Al‐Balqaa University	406 (15.6)
The Hashemite University	457 (17.6)
JUST University	439 (16.9)
Mutah University	457 (17.6)
The University of Jordan	438 (16.8)
The Yarmouk University	405 (15.6)

*Note:* The data is presented as *n* (%) or mean ± stander deviation.

### 3.2 Knowledge levels among participants

The mean knowledge score was 14 (SD ± 2.5) which was out of 20. More than half of the participants (58.84%) demonstrated high knowledge, as indicated by 1531 individuals while the rest had low knowledge 1071 (41.16%). Regarding specific questions, a high percentage of participants answered correctly when asked about the transmission of HBV through sharing injection needles (95.5% correct), the effectiveness of vaccination in protecting against hepatitis B infection (92.0% correct), and the risk of doctors and medical students contracting hepatitis B from patients (90.2% correct). However, the percentage of correct answers was lower for other questions. Specifically, only 40.4% of participants answered correctly when asked about the possibility of contracting HBV from their parents, while even fewer knew that Hepatitis C cannot be spread through kissing or talking (36.6%) and that symptoms do not necessarily appear soon after HCV enters the body (36.0%). Table 2 shows the participants' knowledge scores.

**Table 2:** Participants' Knowledge and practice scores.

Knowledge questions	Corrected answers *n* (%)
Can a person contract (getting infection) HBV from their parents' (heredity)? (No*)	1050 (40.4)
Can coughing or sneezing transmit hepatitis B? (No*)	1894 (72.8)
Can HBV spread through the sharing of injection needles? (Yes*)	2485 (95.5)
Can Hepatitis C be spread through kissing or talking? (No*)	953 (36.6)
Can hepatitis C be transmitted from the mother to her baby? (Yes*)	1977 (76.0)
Can hepatitis C be transmitted via ear or nose piercing? (Yes*)	1467 (56.4)
Can hepatitis C infection cause joint pain? (Yes*)	1927 (74.1)
Can vaccination protect against hepatitis B infection? (Yes*)	2394 (92.0)
Can you protect against hepatitis C infection by vaccination? (No*)	1188 (45.7)
Could hepatitis B infection spread through dialysis? (Yes*)	2218 (85.2)
Do All patients with hepatitis B surface antigen positive need treatment? (No*)	1336 (51.3)
Do the symptoms start to show up soon after HCV enters the body? (No*)	937 (36.0)
Do you believe that doctors and medical students carry the risk of contracting hepatitis B from the patients? (Yes*)	2346 (90.2)
Does Hepatitis B have a link to liver cancer? (Yes*)	2271 (87.3)
Does Nutrition and Exercise help in chronic Hepatitis B treatment? (Yes*)	1623 (62.4)
Is hepatitis C an RNA virus? (Yes*)	2162 (83.1)
Is hepatitis C the most deadly type of hepatitis? (Yes*)	1533 (58.9)
Is there a screening for hepatitis C? (Yes*)	1855 (71.3)
Is there treatment for hepatitis C? (Yes*)	1765 (67.8)
The most common hepatitis is hepatitis B? (No*)	1730 (66.5)
Mean (SD) 14± (2.5)	
**Knowledge levels among participants**	
Low	1071 (41.16)
High	1531 (58.84)
**Practice**	
Are you hepatitis vaccinated (All three dosages)? (Yes*)	1573 (60.5)
Have you ever assessed the level of postvaccination immunity against hepatitis? (Yes*)	967 (37.2)
I will encourage family members to screen for HBV and HCV if a family member is infected by hepatitis. (Yes*)	2334 (89.7)
I will participate in a hepatitis B awareness program. (Yes*)	2193 (84.3)
I will tell All hepatitis C infected people to get treatment. (Yes*)	2301 (88.4)
I will use sterilized syringes when necessary. (Yes*)	2375 (91.3)
I will advise hepatitis B patients not to get pregnant. (No*)	1601 (61.5)
Mean (SD) 4.89± (1.13)	
**Practice levels among participants**	
Poor	910 (35.0)
Good	1692 (65.0)

* The correct answer.

### 3.3 Practice levels among participants

The mean score of practice score was 4.89 (SD ± 1.13) which was out of 7. A large portion of respondents showed good practice 1692 (65.0%) while the rest had poor practice 910 (35.0%). The questions “I will use sterilized syringes, when necessary,” “I will encourage family members to screen for HBV and HCV if a family member is infected by hepatitis,” and “I will tell All hepatitis C infected people to get treatment” were answered correctly by 91.3%, 89.7%, and 88.4%, respectively. On the other hand, only 60.5% and 37.2% answered correctly the questions “Are you hepatitis vaccinated (All three dosages)” and “Have you ever assessed the level of postvaccination immunity against hepatitis,” respectively. Participants' practice scores are presented in Table 2.

### 3.4 Attitude levels among participants

The mean attitude score was 1.6 (SD ± 3.1) which was out of 14. Further, it was observed that a large portion of respondents demonstrated low attitude 2127 (81.7%) while the rest had high attitude 475 (18.4%). Additionally, 59.6% of participants correctly agreed that they can shake hands or hug a hepatitis C‐infected person, and 49.3% of participants correctly agreed that Hepatitis C can be prevented by a healthy lifestyle. However, it was observed that 57.1% of the participants incorrectly agreed with certain statements. Table 3 shows participants' attitude scores.

**Table 3:** Participants' attitude scores.

Attitude	Strongly disagree *n* (%)	Disagree *n* (%)	Neutral *n* (%)	Agree *n* (%)	Strongly agree *n* (%)
HBV and HCV patients should be given the final appointment of the day (The last appointment in the clinic). (True*)	210 (8.1)	526 (20.2)	936 (36.0)	664 (25.5)	266 (10.2)
You can shake hands or hug a hepatitis C infected person. (True*)	201 (7.7)	387 (14.9)	462 (17.8)	1041 (40.0)	511 (19.6)
Hepatitis C can be prevented with immunization and receiving a hepatitis vaccination. (True*)	374 (14.4)	808 (31.1)	461 (17.7)	511 (19.6)	448 (17.2)
Hepatitis C can be prevented by a healthy lifestyle. (True*)	92 (3.5)	425 (16.3)	803 (30.9)	972 (37.4)	310 (11.9)
Liver failure caused by hepatitis B and C infections can be avoided with medications. (False*)	54 (2.1)	361 (13.9)	702 (27.0)	1213 (46.6)	272 (10.5)
Patients who have HBV or HCV should avoid interacting with other family members. (False*)	334 (12.8)	697 (26.8)	531 (20.4)	757 (29.1)	283 (10.9)
Hepatitis B and C are more difficult to get infected than HIV. (False*)	179 (6.9)	508 (19.5)	649 (24.9)	963 (37.0)	303 (11.6)
Mean (SD) 1.6=± (3.1)					
Attitude levels among participants					
Low	2127 (81.7)				
High	475 (18.3)				

* The correct answer.

### 3.5 Subgroup scores

Mann‐Whitney U test and Kruskal‐Wallis tests were employed to evaluate potential differences in means among variables concerning participants' knowledge, attitudes, and practices towards Hepatitis. Regarding knowledge, the analysis revealed a significant difference in knowledge scores according to the gender of respondents, where female (14.1 ± 2.5) respondents had higher mean scores than male respondents (13.8 ± 2.5) (*p* < 0.001, Mann‐Whitney U test). Further, there was a significant difference in knowledge scores based on their academic year; sixth‐year medical students had the highest mean score (15.1 ± 0.1) and first‐year medical students had the lowest (12.2 ± 2.5) (*p* < 0.001, Kruskal‐Wallis test). There was also a significant difference in knowledge scores based on students' universities; students from JUST had the highest mean score (14.3 ± 2.5) while students from Yarmouk University had the lowest (13.42 ± 0.5) (*p* < 0.001, Kruskal‐Wallis test). Furthermore, in terms of attitudes and practices related to Hepatitis, the findings revealed significant variations among participants based on their academic years. Sixth‐year medical students exhibited the highest average score in both attitude (0.4 ± 0.5) and practice (5.4 ± 1.1). While first‐year medical students had the lowest mean score in attitude (0.1 ± 0.4), third‐year medical students displayed the lowest mean scores in practice (4.6 ± 1.1).

**Figure 1:**
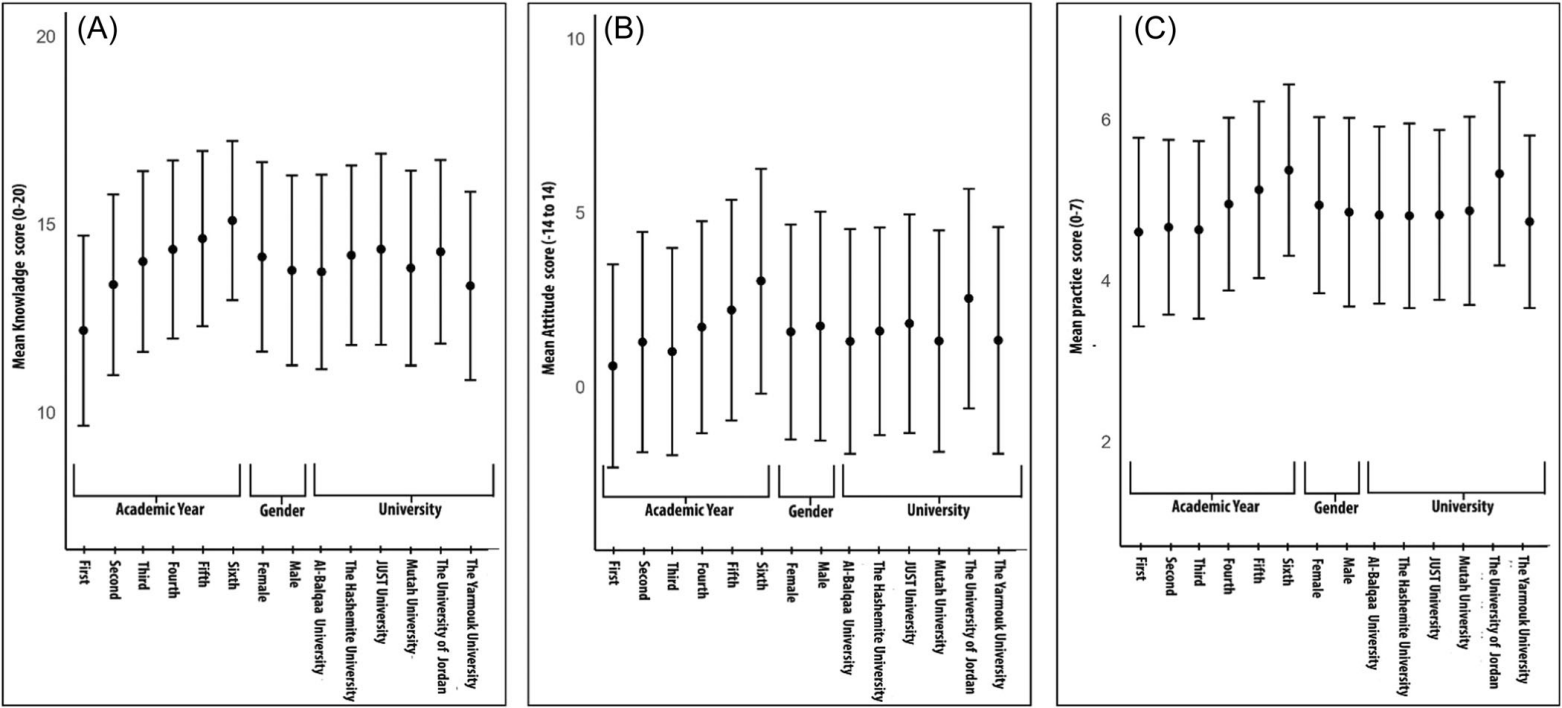
Description of subgroups scores by KAP, (A) Knowledge, (B) Attitude, (C) Practice.

Furthermore, when considering universities, students from the University of Jordan achieved the highest mean scores in attitude (0.4 ± 0.5) and practice (5.3 ± 1.1). Conversely, students from Hashemite University obtained the lowest mean score in attitude (0.2 ± 0.4), while those from Yarmouk University had the lowest mean score in practice (4.7 ± 1.1). These differences were statistically significant with a p‐value of less than (*p* < 0.001, Kruskal‐Wallis test). The study found that there were not any significant differences between males and females regarding their attitude (*p* = 0.2, Mann‐Whitney U test) and practice (*p* = 0.08, Mann‐Whitney U test). Figure 1 depicts subgroup scores for Knowledge, Attitude, and Practice.

### 3.6 Association between demographic characteristics of students and knowledge, attitudes, and practices toward HBV and HCV

A chi‐square analysis was conducted to explore potential variations in knowledge, attitudes, and practices concerning HBV and HCV. Among the respondents, 843 females (55.1%), 328 fourth‐year students (21.4%), and 284 students from The Hashemite University (18.5%) exhibited significantly higher levels of knowledge (*p* < 0.001). Additionally, sixth‐year students 132 (27.8%), and students from The University of Jordan 118 (24.8%) demonstrated significantly elevated levels of attitude (*p* = <0.001). without any significant differences between males and females, (*p* = 0.08). Lastly, fifth‐year students 340 (20.1%) and students from The University of Jordan 346 (20.4%) displayed significantly higher levels of practice (*p* < 0.001). Table 4 demonstrates the association between demographic characteristics of students and knowledge, attitudes, and practices toward HBV and HCV.

**Table 4:** Association between demographic characteristics of students and knowledge, attitudes, and practices toward HBV and HCV (*n* = 2602).

Covariant		Level of knowledge			Level of attitude			Level of practice		
		Low (1071)	High (1531)	*p* value	Low (2127)	High (475)	*p* value	Poor (910)	Good (1692)	*p* value
Age mean (SD)		20.8 (1.8)	21.8 (1.7)	<0.001	21.3 (1.8)	22.0 (1.8)	<0.001	21.0 (1.7)	21.6 (1.9)	<0.001
Gender *n* (%)	Female	525 (49.0)	843 (55.1)	<0.001	1136 (53.4)	232 (48.8)	0.08	458 (50.3)	910 (53.8)	0.1
	Male	546 (51.0)	688 (44.9)		991 (46.6)	243 (51.2)		452 (49.7)	782 (46.2)	
Academic Year *n* (%)	First‐year	285 (26.6)	102 (6.7)	<0.001	349 (16.4)	38 (8.0)	<0.001	170 (18.7)	217 (12.8)	<0.001
	Second‐year	239 (22.3)	223 (14.6)		399 (18.8)	63 (13.3)		204 (22.4)	258 (15.2)	
	Third‐year	157 (14.7)	235 (15.3)		351 (16.5)	41 (8.6)		176 (19.3)	216 (12.8)	
	Fourth‐year	169 (15.8)	328 (21.4)		410 (19.3)	87 (18.3)		158 (17.4)	339 (20.0)	
	Fifth‐year	142 (13.3)	326 (21.3)		354 (16.6)	114 (24.0)		128 (14.1)	340 (20.1)	
	Sixth‐year	79 (7.4)	317 (20.7)		264 (12.4)	132 (27.8)		74 (8.1)	322 (19.0)	
University *n* (%)	The Hashemite University	173 (16.2)	284 (18.5)	<0.001	381 (17.9)	76 (16.0)	< 0.001	182 (20.0)	275 (16.3)	<0.001
	JUST University	158 (14.8)	281 (18.4)		352 (16.5)	87 (18.3)		168 (18.5)	271 (16.0)	
	Mutah University	195 (18.2)	262 (17.1)		387 (18.2)	70 (14.7)		159 (17.5)	298 (17.6)	
	The University of Jordan	158 (14.8)	280 (18.3)		320 (15.0)	118 (24.8)		92 (10.1)	346 (20.4)	
	The Yarmouk University	200 (18.7)	205 (13.4)		342 (16.1)	63 (13.3)		159 (17.5)	246 (14.5)	
	Al‐Balqaa University	187 (17.5)	219 (14.3)		345 (16.2)	61 (12.8)		150 (16.5)	256 (15.1)	

Abbreviations: *n*: number; SD, standard deviation.

### 3.7 Multivariable analysis of knowledge, attitude, and practice

In terms of predictive factors of high knowledge, attitude, and practice, a multinomial regression model revealed that male respondents were less likely to have a high knowledge compared to female respondents (OR: 0.73; *p*: 0.001; 95% CI: −0.50 to −0.13). Further, students in the first to fifth year were less likely to have a high score compared to sixth‐year students. Additionally, it was observed that students enrolled at JUST were notable as significant positive factors for possessing high knowledge (OR: 1.6; *p* = 0.003; 95% CI: 0.16–0.80) and high attitude (OR: 1.61; *p* = 0.03; 95% CI: 0.06–0.89). On the other hand, students attending the University of Jordan emerged as significant positive predictors for having a high attitude (OR: 2.77; *p* < 0.001; 95% CI: 0.60 to 1.44) and high practice (OR: 1.54; *p* < 0.001; 95% CI: 0.28 to 0.58). Results of multivariable analysis of the knowledge, attitude, and practice are demonstrated in Table 5.

**Table 5:** Multivariable analysis of the knowledge, attitude, and practice.

Covariant	Knowledge			Attitude			Practice		
	OR	CI	*p* value	OR	CI	*p* value	OR	CI	*p* value
Age	0.92	−0.17 to 0.01	0.09	0.98	−0.13 to 0.10	0.79	1.03	−0.01 to 0.07	0.11
Gender	—								
Male	0.73	−0.50 to −0.13	0.001*	1.17	−0.08 to 0.40	0.20	0.92	−0.16 to 0.01	0.08
Female	Reference			Reference			Reference		
Academic Year	—								
First‐year	0.039	−3.79 to −2.72	<0.0001*	0.09	−3.11 to −1.72	<−0.00001*	0.57	−0.81 to −0.32	<0.0001*
Second‐year	0.13	−2.48 to −1.57	<0.0001*	0.17	−2.39 to −1.20	<−0.00001*	0.55	−0.81 to −0.39	<0.0001*
Third‐year	0.26	−1.76 to −0.94	<0.0001*	0.13	−2.54 to −1.47	<−0.00001*	0.54	−0.81 to −0.43	<0.0001*
Fourth‐year	0.38	−1.33 to −0.59	<0.0001*	0.29	−1.72 to −0.77	<−0.00001*	0.76	−0.45 to −0.11	0.001*
Fifth‐year	0.55	−0.92 to −0.27	<0.0004*	0.43	−1.27 to −0.41	0.0001*	0.82	−0.34 to −0.04	0.01*
Sixth‐year	Reference			Reference			Reference		
University	—								
The Hashemite University	0.99	−0.33 to 0.33	1	1.02	−0.41 to 0.44	0.94	0.86	−0.30 to 0.00	0.05
JUST University	1.6	0.16 to 0.80	0.003*	1.61	0.06 to 0.89	0.03*	0.96	−0.18 to 0.12	0.67
Mutah University	1.02	−0.30 to 0.33	0.91	1.00	−0.41 to 0.42	0.98	1.01	−0.13 to 0.16	0.87
The University of Jordan	1.29	−0.06 to 0.58	0.11	2.77	0.60 to 1.44	<0.001*	1.54	0.28 to 0.58	<0.0001*
The Yarmouk University	0.96	−0.37 to 0.29	0.811	1.16	−0.28 to 0.58	0.50	0.96	−0.19 to 0.11	0.63
Al‐Balqaa University	Reference			Reference			Reference		

Abbreviations: CI, confidence interval; OR, odd ratio.

* Significant *p* value.

### 3.8 Spearman's correlation between the knowledge, attitude, and practice scales

Spearman's correlation was conducted to assess the association between the knowledge, attitude, and practice scales. As shown in Table S1, there was a significant moderate positive association between knowledge and attitude (*r*: 0.33, *p* < 0.001). Further, there were significant weak positive associations between knowledge and practice on one side (*r*: 0.17, *p* < 0.001) and attitude and practice from the other side (*r*: 0.133, *p* < 0.001).

## 4 DISCUSSION

The study evaluates the knowledge of medical students regarding hepatitis, uncovering interesting findings. About 58.84% demonstrated a high level of knowledge, suggesting a solid understanding crucial for their future roles in healthcare and indicating their preparedness to offer accurate information and effectively implement preventive measures. Nonetheless, there are knowledge gaps, with 1071 students (41.16%) showing a low level of knowledge, emphasizing the need for focused educational interventions. Variances in educational systems, cultural contexts, and sample sizes contributed to disparities among studies. In the Moroccan study,^15^ 31.1% demonstrated high knowledge, 48.6% moderate, and 20.2% low. Ghana's public health students exhibited 17.3% good, 73.9% moderate, and 8.9% poor knowledge.^16^ Vietnamese studies revealed considerable differences: 89.2% of medical students had good knowledge^13^ (Hepatitis B), whereas another study showed 74.4% good knowledge (Hepatitis C) and only 3.2% with a good attitude.^17^ Syrian medical students displayed 59.3% average and 23% good knowledge.^18^

Educational variations across countries likely influenced disparities. Cultural attitudes towards health, distinct healthcare accessibility, and differing emphases on specific diseases in education may have impacted knowledge levels. Sample sizes also played a crucial role; larger and more diverse samples provided a comprehensive understanding. The studies' focused approach some targeting specific virus types might explain differing knowledge distributions. A comprehensive, standardized methodology across regions and comparative analyses of educational systems could deepen our understanding. Such insights could aid in tailored educational interventions and effective public health initiatives for diverse student populations.

A closer look at the data reveals specific areas of lower knowledge, such as hepatitis B transmission (40.4%) and misconceptions about hepatitis C (36.6% knew it doesn't spread through casual contact). These findings emphasize the need for focused education to rectify misconceptions. Compared with prior studies, a recurring pattern of moderate knowledge levels among medical students is observed,^19^ highlighting the ongoing need for curriculum improvements.^20^ Gender disparities in knowledge levels, with males scoring lower,^21^ further stress the importance of addressing these gaps for equitable educational outcomes.^22^

A substantial majority of participants, approximately (65.0%), reported a good level of practice regarding Hepatitis, which is indicative of a positive attitude towards adopting preventive measures and maintaining healthy practices. This high percentage reflects a commendable commitment to public health among the study population.^11^ However, it is noteworthy that around (35%) of participants reported a poor level of practice. While this group is smaller, it still represents a significant portion of the sample and presents an opportunity for targeted interventions. Understanding the specific reasons for their moderate practices and tailoring educational or awareness programs to address their needs could further improve overall Hepatitis prevention efforts.^23^ On a positive note, the study found a low percentage (35%) of participants reporting low practice levels, suggesting that, on the whole, the study population exhibited good practices related to Hepatitis. This is encouraging and suggests that public health campaigns and educational initiatives related to Hepatitis have made substantial progress in promoting responsible healthcare practices within the community.^24^, ^25^ Moreover, the high percentages of correct answers for specific practice questions, such as using sterilized syringes and encouraging family members to screen for Hepatitis, indicate a strong inclination toward responsible healthcare practices.^26^ This highlights the success of awareness programs in disseminating important information and encouraging positive behavior change.^27^

Significantly, the study findings indicate that a substantial majority of respondents, comprising 81.7%, reported a low level of attitude towards Hepatitis. This implies that most participants exhibited a less favorable stance, potentially hindering effective Hepatitis awareness and prevention efforts.^28^ Nevertheless, this less favorable outlook could serve as a solid foundation for future initiatives aimed at enhancing awareness and prevention, as identified by Hilleman.^28^ On a positive note, it is noteworthy that 18.3% of participants demonstrated a high level of attitude, reflecting a positive and proactive stance towards Hepatitis. This subgroup could be viewed as potential advocates for Hepatitis awareness campaigns and initiatives, given their evident enthusiasm, as suggested by FitzSimons et al.^29^ The prevalence of such a positive disposition signifies a promising starting point for public health efforts focused on Hepatitis, aligning with the findings of Afihene et al.^30^ However, the study's results also highlight areas that require improvement, particularly in addressing certain misconceptions. It is crucial to address these gaps to ensure a more comprehensive and effective approach to Hepatitis awareness and prevention.

The finding that (57.1%) of participants incorrectly agreed with specific attitude statements indicates the presence of gaps in knowledge and understanding. Addressing these misconceptions through targeted education and awareness programs can play a vital role in strengthening public attitudes and beliefs towards Hepatitis prevention and management.^31^ Moreover, the identification of significant disparities in attitudes and practices based on academic years and universities emphasizes that these factors also play a pivotal role in shaping individuals' attitudes and behaviors concerning Hepatitis.^32^ This insight underscores the importance of tailoring educational and awareness campaigns to address the unique needs and challenges faced by students in various academic settings.^33^

Furthermore, the multinomial regression model's revelation that male respondents were less likely to possess high knowledge compared to their female counterparts is a noteworthy finding. It points to potential gender‐related disparities in Hepatitis knowledge, signaling the necessity for focused interventions aimed at bridging this gap.^34^ Addressing these disparities can help ensure more equitable access to essential Hepatitis‐related information and resources across genders 30.^35^

The identified knowledge gaps and disparities in attitudes and practices among Jordanian medical students have far‐reaching implications. First, they call for immediate curricular adjustments in medical schools to enhance hepatitis‐related education. Integrating targeted modules or workshops focusing on key areas of deficiency, such as transmission mechanisms and dispelling misconceptions, is crucial. These findings also underscore the urgency of reevaluating existing public health policies related to hepatitis prevention and management. Tailored interventions in educational settings and broader public health campaigns are warranted to address these gaps effectively.

Based on these findings, interventions can take multiple forms. Implementing innovative teaching methodologies, incorporating interactive sessions, and utilizing technology‐driven educational tools could enhance knowledge acquisition among students. Collaborative efforts between academia, healthcare institutions, and public health authorities are essential in designing comprehensive programs. Further research should delve deeper into the efficacy of specific educational interventions in bridging knowledge gaps and reshaping attitudes and practices. Longitudinal studies tracking the impact of revised curricula or targeted interventions could offer invaluable insights into sustained behavioral changes.

To our knowledge, this study which aims to investigate the knowledge, attitude, and practice of medical students towards hepatitis B and C, has the biggest sample size from all medical schools in Jordan. Thus, it gives it more potential regarding its ability to generalize the results. On the other hand, despite its advantages and strengths, the cross‐sectional study was based only on participant self‐reporting and the inability to verify. Furthermore, it is impossible to draw any cause‐effect relation. Also the study did not include discrimination scores for internal and external validity, as it was not designed to develop a predictive or assessment tool. This shall limit our ability to assess how effectively the tool distinguishes between varying levels of knowledge (attitude and practice) and the applicability across different contexts. While the study focused on face and content validity and confirmed reliability through Cronbach's alpha, the absence of discrimination scores affects the evaluation of the tool's overall performance. To address this limitation, future research should include discrimination scores to better assess the tool's ability to differentiate and generalize the effectiveness in diverse settings.

## 5 CONCLUSION

In conclusion, this study of 2602 participants' knowledge, attitudes, and practices about hepatitis B and C revealed a wide demographic profile with a range of medical education levels. Participants showed a range of views, high adherence to some practices, and intermediate knowledge levels. Gender, academic year, and university affiliation were identified as significant variables by subgroup analysis and association. The importance of these factors on knowledge, attitudes, and practices was shown using multivariate analysis. There were associations between practices, attitudes, and knowledge. These revelations highlight the necessity of focused interventions to close gaps, support healthy behaviors, and foster educated health perspectives for a healthier future.

## AUTHOR CONTRIBUTIONS

**Husam Abu Suilik**: Conceptualization; methodology; formal analysis; writing—original draft; writing—review and editing; data curation. **Osama Alfaqeh**: Methodology; data curation; writing—original draft; writing—review and editing. **Abdalqader Alshrouf**: Methodology; data curation; writing—original draft; writing—review and editing. **Omnia M. Abdallah**: Methodology; data curation; writing—original draft; writing—review and editing. **Jehad Feras AlSamhori**: Methodology; data curation; writing—original draft; writing—review and editing. **Lujain Lataifeh**: Methodology; data curation; writing—original draft; writing—review and editing. **Mohammad Alsbou**: Methodology; data curation; writing—original draft; writing—review and editing. **Mohammad Abusuilik**: Methodology; data curation; writing—original draft; writing—review and editing. **Nawar Khalil**: Methodology; data curation; writing—original draft; writing—review and editing. **Mohand Odeh**: Methodology; data curation; writing—original draft; writing—review and editing. **Hashem Abu Serhan**: Writing—original draft; writing—review and editing. **Abdulqadir J Nashwan**: Writing—original draft; Writing—review and editing. **Anas Bani Hani**: Methodology; data curation; writing—original draft; writing—review and editing; supervision.

## CONFLICT OF INTEREST STATEMENT

The authors declare no conflict of interest. Abdulqadir Nashwan is an Editorial Board member of Health Science Reports and a coauthor of this article. To minimize bias, they were excluded from all editorial decision‐making related to the acceptance of this article for publication.

### 1 ETHICS STATAEMENT

The study was conducted according to the guidelines of the Declaration of Helsinki and approved by Institutional Review Board (IRB) at the Hashemite University in Zarqa, the Hashemite Kingdom of Jordan, on 26 December 2022 in meeting No. 2200834.

## TRANSPARENCY STATEMENT

The lead author Abdulqadir J. Nashwan affirms that this manuscript is an honest, accurate, and transparent account of the study being reported; that no important aspects of the study have been omitted; and that any discrepancies from the study as planned (and, if relevant, registered) have been explained.

## Supporting information

Supporting information.

Supporting information.

## Data Availability

The data that support the findings of this study are available from the corresponding author upon reasonable request.
