# Supplementary material for: Medical students' knowledge, attitude, and practice regarding hepatitis B and C virus infections in Jordan: A cross‐sectional study
Source: Health Sci Rep. 2024 Dec 11;7(12):e70150. doi: 10.1002/hsr2.70150 (PMC11635120; doi:10.1002/hsr2.70150)
Supplement: Supplementary file 2 — Supporting information. [file HSR2-7-e70150-s001.docx]

**Table S1:** Knowledge, Attitude, and Practice correlation.

| Domain | knowledge | Attitude | Practice |
| --- | --- | --- | --- |
| Knowledge | 1 | 0.33 | 0.17 |
| Attitude | 0.33 | 1 | 0.133 |
| Practice | 0.17 | 0.133 | 1 |
